# What Is Wrong with Solidarity in EU Asylum and Migration Law?

**DOI:** 10.1007/s42439-022-00059-4

**Published:** 2022-05-04

**Authors:** Eleni Karageorgiou, Gregor Noll

**Affiliations:** 1 Faculty of Law, Lund University, Lund, Sweden; 2 Department of Law, University of Gothenburg, Gothenburg, Sweden

**Keywords:** Solidarity, EU, Asylum, Migration, Alliance, EU pact

## Abstract

In this article, we explore why solidarity has not worked according to expectation in EU migration and asylum law and why it is unlikely to work in the future. First, we consider discourses of burden-sharing and solidarity in EU law from the 1990s up to the Lisbon Treaty in 2009 to identify emergent path dependencies. This period saw the introduction of primary law provisions on solidarity, such as Article 80 TFEU, as French and Dutch electorates had rejected a European constitution. Second, we perform an analysis of Article 80 through the conceptual history of solidarity, in particular, the dominant Roman law tradition of obligation in solidum and the French tradition of solidarism. We submit that the term ‘solidarity’ is actually a misnomer: already on structural grounds, Article 80 should be read as an alliance clause, countering a threat of irregular immigration. Third, we find that the practice under Article 80 as it develops during the period between 2015 and the 2020 European Commission Pact on Migration and Asylum corroborates this finding. Overall, we find that the concept of solidarity in EU asylum and migration law engenders outcome expectations that it cannot deliver as the defence alliance it is.

## Introduction

In September 2020, the European Commission released the new Pact on Migration and Asylum (henceforth ‘the Pact’)^1^ initiating a set of legislative proposals for the long-awaited reform of the so-called Common European Asylum System (CEAS). In an effort to address the reception and solidarity crisis of 2015, the Commission envisages a ‘flexible yet mandatory’ approach to the way in which responsibility is allocated among the Member States with a view to ‘closing the existing implementation gap’ and building trust across the Union.^2^

The Pact has been the subject of extensive analysis by commentators.^3^ It is widely seen as a repackaging of old tricks rather than a fresh start, as it retains the focus on border intensification (by pre-screening), continues to put pressure on the periphery (by enhanced border procedures in combination with Dublin’s first entry rule) and pursues externalization (through return sponsorship). Broadly speaking, there are four main points of critique against the Pact’s approach to solidarity. First, the new proposals do not envisage an overhaul of the Dublin system’s main rules and underlying rationale. Second, the new ‘flexible’ solidarity mechanism entails a complex combination of voluntary pledges and mandatory contributions institutionalizing a wide range of solidarity actions ranking from relocation to capacity building,^4^ contrary to a vision of solidarity as undifferentiated responsibility for all, reflected in the 2015 Relocation Decisions^5^ and reaffirmed by the Court of Justice of the EU (CJEU) in the *Slovakia and Hungary v Council* case.^6^ Third, the Commission’s additional powers are called into question, as it supposedly carries out a series of assessments and corrective adjustments should pledges by Member States fall short of asylum demands in times of migratory pressure. Fourth, the ‘hybridization’ of distinct or separate EU and national policy domains (asylum, migration enforcement and border management) reflected in the Pact’s ‘seamless link’ has been deemed as its most problematic feature, because it taints refugeehood with irregularity and covertly shifts the focus to externalization through return sponsorship and other measures.^7^

Evidently, commentators have addressed *what* will not work in the new proposals so as to engender solidarity in EU migration and asylum law. In this critique, the concept of solidarity is often taken for granted. By contrast, our article seeks to explore *why* solidarity neither has worked nor will work according to expectation in EU migration and asylum law. We answer this question by relating solidarity in the contemporary history of EU law to pivotal points in the conceptual history of solidarity. In Sect. 2, we shall first map solidarity in the history of EU migration and asylum law to explore which of these concepts is most relevant for its development. In Sect. 3, we will explore different conceptions of solidarity in legal history and contrast them with the competing concept of the alliance. In Sect. 4, we shall turn to a detailed analysis of cooperation structures in the 2020 Pact and establish an argument why the Pact effectively thwarts the expectations that solidarity language entails. Sect. 5 offers conclusions.

## Solidarity in the History of EU Integration on Migration and Asylum

### The 1990s: Large-Scale Refugee Movements and the Need for Burden-Sharing

During the 1990s, a discourse on ‘burden-sharing’ was at the core of the discussions relating to the creation of a common European asylum system, involving both EU Member States and non-members. However, the decade also saw the emergence of a separate discourse on solidarity, which was about offsetting the distributional effects of the 1990 Dublin Convention, determining which state would be responsible for processing an asylum claim in substance. It is important to track burden-sharing and solidarity as separate concepts to understand how the thinking behind them informed choices in later periods.^8^

It was, in fact, the failure of the 1990s normative infrastructure to respond to the large refugee movements from Bosnia and later Kosovo that triggered a heated debate on burden-sharing in the region. Unlike the response to the Bosnian crisis, which was limited to funding for humanitarian relief to the geographically most proximate countries, the Balkan crisis did trigger a rather organized evacuation response. Despite the large-scale responsibility sharing it had mobilized in western Europe and around the world, the Humanitarian Evacuation Programme (HEP) has been criticized for exacerbating the uneven distribution of spontaneous arrivals existed from the start of the Kosovo exodus by increasing the load of those EU Member States who had been receiving both individual refugees from Kosovo and beneficiaries through HEP.^9^ Arguably, the Kosovo case is indicative of the fact that ad hoc sharing does not necessarily guarantee a fair allocation of responsibilities. The term ‘burden-sharing’ appeared to be inspired by debates on the uneven distribution of efforts within NATO ever since the 1970s. The debate on European asylum burden-sharing took place in a militarized context, where asylum seekers’ movements were discussed alongside armed interventions and diplomatic mediation efforts on the Balkans.

Although prominent, these discussions did not provide fertile ground for anything more than vague commitments, even in the case of the refugee movements from the former Yugoslavia. Rather, the idea was to contain the flight from the conflict to the region in distress and relief efforts have for the most part been carried out by the UNHCR and by NGOs. Although in resolutions passed in 1992 and 1994 by the EP and in 1995 by the Council it was recognized that the relocation of persons from the crisis regions should be prioritized,^10^ existing norms of containment based on safe third-country considerations permeated asylum policies of the Member States.^11^

With the parallel development of the Schengen rules, however, cooperation in the field of asylum policy in the EU was guided by a notion of solidarity faithful to the internal market. Through the exclusion of the refugee from free movement, solidarity was directed towards a core of Member States’ populations who were seen as threatened by irregular movements after the abolition of internal borders. This logic has eventually determined the rationale of the systems allocating responsibility established through the Dublin framework.^12^ At the end of the 1990s, two baseline conceptualizations were in place, with ‘burden-sharing’ relating to asylum inflows in situations of crisis and ‘solidarity’ signposting the future resolution of distributional inequality inherent in the Dublin Convention. If ‘burden-sharing' was about managing an on–off external threat, ‘solidarity’ was to iron out the deficiencies accumulating in the routine operation of the Dublin system.

### The Amsterdam Treaty and the Need to Revisit Earlier ‘Political Choices’

In the late 1990s, the need for an instrument that does more than responding to a particular set of events such as the Bosnia and Kosovo crisis had been considered crucial. This was reflected in the major institutional achievement towards the communitarization of asylum and migration policy, namely, the adoption of the Amsterdam Treaty which entered into force in 1999 and introduced solidarity as a guiding norm of the EU asylum policy, by prescribing ‘a balance of effort between Member States in receiving and bearing the consequences of receiving refugees and displaced persons’.^13^ Along the same lines, the Tampere conclusions emphasized solidarity between the Member States, primarily, in relation to temporary protection.^14^

Interestingly enough, the Treaty of Amsterdam did identify a link between free movement of persons in an area without internal frontiers and related flanking measures on criteria for assigning responsibility for asylum applicants (Art. 61a TEC). As stressed by the Commission though, the treaty does not state ‘whether there should be a link between the duty to control the external border and the allocation of responsibility for an asylum applicant’.^15^ That was ‘a political choice’ made by the drafters of the Dublin Convention which, according to the Commission, should be re-assessed in future reforms in view of implementation challenges.^16^ At that point, replacing the Dublin Convention with a mechanism for distributing asylum applicants between the Member States in proportion to each Member State’s capacity to receive them was not considered as a ‘pragmatic’ solution, particularly since discussions on physical burden-sharing had not produced any concrete results.^17^

Along the same lines, in the discussions preceding the adoption of the Temporary Protection Directive, it was emphasized that concrete solidarity measures as a response to a mass influx should materialize mainly through financial assistance and as a subsidiary means, through the distribution between the Member States of people granted temporary protection.^18^

The Council Decision establishing a European Refugee Fund (ERF)^19^ which complemented the 2001 Temporary Protection Directive to give effect to financial solidarity was the first binding instrument of the first phase of the EU asylum legislation. Unlike the 2020 Pact discussed in detail in Sect. 4, the Directive did not include a specific obligation for states to take refugees or asylum seekers from each other. However, the flexibility component where states indicate their capacity for refugee reception followed by a negotiation on the number to be received by each state seems to remain. Pursuant to the Directive, solidarity with Member States in the support of their reception efforts is to be shown, firstly, through the allocation of financial resources from the ERF and, secondly, through the distribution of displaced persons among Member States on a voluntary basis and with their agreement. What most commentators have identified as the main challenges for the Directive’s implementation was the challenge in agreeing on whether a situation of ‘mass influx’ exists, the lack of a sufficiently firm and mandatory solidarity commitment and the ‘protection-oriented’ character of the Directive, which was seen as a pull factor, thus making EU states reluctant to apply it.^20^ The first two challenges, namely, the assessment of there being a situation of pressure and the lack of sufficiently clear deliverables by the Member States, have not been addressed by the Pact. At the same time, the obligatoriness for Member States to commit to solidarity measures does not guarantee compliance. As the case of the 2015 emergency relocation mechanism has shown, even in cases where a mass influx has been declared and a mandatory quota system—less attentive to protection standards—was introduced, there were still challenges relating to the system’s application and justiciability.

Given that European states were fundamentally reluctant to commit to burden-sharing at the time, harmonization of policies on border management was considered critical for the development of a common European approach to asylum; the impact of border crossing on the way in which asylum responsibilities are allocated among the Member States was crystal clear. A security conception of migration and asylum influenced by the ‘War on Terror’ climate following 9/11 and the terrorist attacks in Spain (2004) and the UK (2005) has shaped the EU policy agenda as reflected in the Hague Programme.^21^ Despite stating that the Programme aimed at striking the right balance between immigration law, enforcement purposes and fundamental rights protection, nonetheless, it clearly prioritized the dimension of security.

In light of the above, solidarity in this early phase of the CEAS sought to materialize through sharing of financial resources (the creation of the ERF), of norms (the adoption of the first Directives on asylum) and of operational support (the adoption of the 2004 Regulation establishing a European Agency for the Management of Operational Cooperation at the External Borders, Frontex Regulation).^22^ The establishment of the Agency, assisting Member States with implementing the operational aspects of external border management, including return of third-country nationals illegally present in the Member States, has been seen as an essential step towards a well-functioning CEAS. The predominant view was that any solidarity initiative was to respect and complement the rules of responsibility established by the Dublin Convention. Equally, there seems to have been a consensus that refugees should remain either in their countries of origin (internalization) or in the neighbouring region (containment).^23^ In particular, elaborating rules on the basis of which an asylum application would not be considered on its merits by EU Member States but rather shifted to a third country has been seen as way ‘to reduce pressure on asylum determination systems’ that were ‘excessively burdened’.^24^

The end of the first phase of the CEAS coincided with the Union’s Eastern enlargement with particular implications for European integration. In May 2004, ten new Member States joined the Union strengthening its role in the region. As it will be explained in the following paragraph, enlargement meant that the Union would have to address various and often competing accounts of solidarity in the migration context brought by its Eastern allies.

### Lisbon Marking the CEAS ‘Honeymoon’ Phase

The supranationalization of rules on asylum and migration in the 2009 Lisbon treaty brought solidarity into primary law. It constitutionalized the objective for achieving a common policy on migration and asylum, based on solidarity and fairness towards third-country nationals, in accordance with the Charter of Fundamental Rights of the EU.^25^ Henceforth, Article 80 TFEU would be the touchstone for ongoing debates centred on solidarity:

The policies of the Union set out in this Chapter and their implementation shall be governed by the principle of solidarity and fair sharing of responsibility, including its financial implications, between the Member States. Whenever necessary, the Union acts adopted pursuant to this Chapter shall contain appropriate measures to give effect to this principle.

By separating ‘the principle of solidarity’ from the ‘fair sharing of responsibility’, Article 80 TFEU replicates the division into burden-sharing and solidarity that had emerged during the 1990s. The ‘principle of solidarity’ related to the ordinary, daily operation of the CEAS, while the notion of ‘fair sharing’ addressed extraordinary situations of crisis. As the ordinary operation of the CEAS featured more and more crisis elements in the decade following Lisbon, this separation appears less plausible to us today.

The incorporation of the principle of solidarity in EU primary law generated expectations that alternative approaches to up until then unfair and unworkable political choices would be re-assessed in a principled manner. However, the EU’s political priorities seem to have remained unchanged. In the 2009 Stockholm Programme, the European Council referred to ‘the development of a forward-looking and comprehensive Union migration policy, based on solidarity and responsibility’, albeit in a manner that resembled earlier approaches; controlling the external borders, combating illegal migration, building up partnerships with third countries and developing effective return policies.^26^ In the following section, we reflect on what kind of expectations the usage of the solidarity language in European asylum law and policy generates, based on two dominant solidarity traditions, namely Roman Law solidarity and French 19th century solidarism.

To be sure, these traditions do not exhaust the accounts of solidarity throughout history. We draw on them here as paradigmatic instances from what is a much more variegated historical field and as traditions that are considered as the foundations of the idea of Europe and later of the European Union.  

## Reading Article 80 TFEU Through Different Conceptions of Solidarity

### A Conceptual History of Solidarity in Law

As we saw, Article 80 TFEU concerns solidarity and fair sharing between Member States only and not solidarity between Member States and refugees or Member States and other recipient states in crisis regions. Historically, ‘solidarity’ as a legal term of art describes a quite different relationship. We will argue that the term ‘solidarity’ in Article 80 TFEU is simply out of place and risks creating false expectations. Which is exactly what has happened in the refugee reception crisis of 2015 and in the discourse on the 2020 Pact on Migration and Asylum.

While the concept of solidarity has engendered a vast literature,^27^ we believe that tracking it back to the reception of Roman law is particularly worthwhile. Obligation in solidum is a nineteenth-century concept based on the Roman law of obligations and its techniques to provide personal security to a creditor. Rather than merely relying on the debtor, the creditor may turn to another person (called the surety) who bound himself to that creditor as being responsible for the fulfilment of the debtor’s obligation. Think of a group of actors assuming this obligation in solidum, and you have a pretty robust construction for getting back what you are owed. This particular technique lives on in all modern legal systems.^28^

The most favourable form of a Roman surety transaction comes without any restrictions on the amount which the creditor may demand from any one of the sureties.^29^ This is the most solid personal security one may think of under Roman law, and it gave rise to the concepts of obligation in solidum and of solidarity. How the debtors among themselves handle the situation obtaining when the creditor has demanded payment from one of them and is a secondary question that has been dealt with separately both in Roman law and its reception in civil law systems. Evidently, the smooth functioning of obligation in solidum depends on a well-designed agreement among the sureties on how to respond to it or on general legislation.^30^

Up and until 1835, the German sociologist Rainer Zoll tells us, this understanding of solidarity as surety prevails to the degree that the *Dictionnaire de l’Academie francaise* defined it *only* in this way, emphasizing that solidarity may not be presumed but needs to be declared explicitly.^31^ What is rendered *solid* in this construction is the likelihood of the legal obligation being honoured. This is done by extending the personal base and by unifying it in obligation. To the creditor, the debtor and the sureties become all one. Here is a feature explaining why the solidarity concept is so attractive for later appropriation, first by the French solidarists and then by the European Union. While consisting of sovereign states, the EU wishes to appear as an inseparable unit to outsiders as much as to itself.

So, the *Dictionnaire de l’Academie francaise* of 1835 still knew only of Roman legal solidarity. Then something happened: as an outflow of the revolution, French nineteenth-century scholars looked for a positivist base of the law in the social. As it happened, they converged on a reconceptualization of solidarity, doing away with the crispness and clarity the term possessed in the Roman law of obligations. French solidarism was designed as a compromise offer by the bourgeoisie to quell revolutionary predilections by those most exposed to the maelstrom of industrialization.^32^ In the long run, the work of French positivists, duly backed by the Holy See,^33^ provided for the reception of solidarity into the doctrines of French public law. All this crystallizes in the preamble of the French Constitution of 1948 which reads ‘La Nation proclame la solidarité et l’égalité de tous les Français devant les charges qui résultent de calamités nationales’. (The nation proclaims the solidarity and equality of all French as to the charges resulting from national calamities.)

From the vantage point of Roman law, that is quite a shift. Is solidarity used to describe the relationship between creditor and a surety? The only creditor candidate would be *les calamités nationales*, in which case *tous les Francais* would be debtors and sureties. As it is characteristic of calamities that they do not obey human laws, this would not make much sense. Rather, the usage of solidarity in this provision moves us into what would have been a secondary issue in Roman law: the relationship *between* the French people as the sureties of the national calamity. So, what it does is to denote the relationship between *tous les Francais* as creditors (meaning those primarily hit by national calamities) and *tous les Francais* as debtors (meaning those not being primarily affected). The arbiter of this process is *la Nation*. This is French positivist solidarity, which focuses on a contractual relationship between those taking a loss caused by an outsider.

One more word on why nineteenth-century French scholars felt they needed solidarity. Imagine that the revolution was the defining event of your epoch. As you are interested in *laicité*, you will very likely come to be interested in positivism. You will find the idea of a social contract problematic, even if it was something of the official doctrine at the time, because there is no positive trace of anyone signing any such social contract. You will look for an alternative to that social contract idea. In which case you might be Alfred Fouillée (1838–1912) and you might believe that the state is an organism, inspired by the contemporary writings of Spencer and Darwin.^34^ Why does this help you to be a positivist? The people and their social relations, you would think, are real, whereas the signing of a social contract is just an idea. The interdependence in social relations is such that it functions as a quasi-contract. If the workers, for example, are not paid in parity to their actual contribution to the survival of the social organism, *une dette social*, a social debt emerges.

With our historical distance, we see how Fouillée was both faithful to positivist thinking (the state being a factual organism) and violated its precepts (this organism being a quasi-contract, a relapse into metaphysics). Emile Durkheim took this further by jettisoning the idea of a quasi-contract. He elevated solidarity to be so indispensable for the people’s survival that freedom were to be subordinated to it. Its moral normativity, he believed, expressed itself in law. Does this give us a hint why Article 80 TFEU was deemed necessary? Were the drafters of the Lisbon Treaty anxious that the moral imperative of solidarity was not sufficiently expressed in the law yet? A late monument to Durkheimian thought?

The story does not end here: solidarism would have its most influential promoter in Léon Duguit. In line with Durkheim, he casts solidarity as a factual condition for human life, and in that, solidarity demands behaviour in conformity with it. ‘La notion de solidarité implique la conception d’une règle de conduit, suffisante pour déterminer les pouvoirs et les devoirs de l’homme social’.^35^ We see him tucking back any sense of prescription into sheer positivist description: a norm of solidarity is ‘implied’ in the fact of solidaristic human life. Duguit’s solidarity is a social law, and this law has to be obeyed not because it is good or beneficial, but, as he put it laconically, ‘because it is’.^36^

Why would the French be so preoccupied with solidarity? A simple answer would be that solidarity might attract those whose frustrated hopes for fraternity would otherwise lead them into the arms of socialism. If a society’s laws are in conflict with solidarity, it seems, solidarity is to be obeyed as any law of nature would be obeyed. Whether you hope for evolution of the laws or their abolition through revolution, French solidarity thinking would keep you away from the more radical position of class struggle taken by the socialists.

Leaping forward to the interwar period, with fresh examples of *la dette social* and the additional burdens of war resulting in revolutions across Europe, we can trace how French solidarism morphed into an internationalized form. The Locarno Pact and its promise of peace and security in the region (usually referred to as the Locarno spirit) led to the belief that a policy of cooperation between European countries would be the way forward. Ideas pointing to ‘a United States of Europe’ or ‘pan-Europe’, suggesting a federation not affecting the sovereignty of the participant nations, were put forward by leading political figures at the time, including Richard von Coudenhove-Kalergi, Aristide Briand and Nicolas Politis whose vision was informed by French solidarism and the French sociological school to international law.^37^ Coudenhove-Kalergi was the founder of the International Pan-European Union, the first and best known of the Europeanist groups to emerge after 1918, and he put forward an idea for European Union from the liberal democratic or bourgeois perspective. His proposal aimed at offering a way out of the great problems that confronted Europe in the years after the peace settlement, namely, the incapacity of democratic regimes to deal with the danger of European war, the risk of terrible economic collapse and the threat of Bolshevism.^38^ Building a security alliance through cooperation internally and antagonism externally—directed towards either or both of the emerging superpowers, the USA and Russia, has been the driving force of what later on became a juridical and economic European Union.^39^ The antirevolutionary features of French solidarism, originally playing out on the level of French class society, had now assumed a geopolitical dimension.

Largely, this leaves us with two analytic frameworks: that of Roman legal solidarity and that of French public law solidarity and its later derivative. Roman legal solidarity is triggered by an intra-contractual event and therefore quite predictable: a creditor calls on one of the sureties to honour her obligation. French public law solidarity as manifested in the 1948 Constitution’s preamble is constituted by a calamity: beyond all form of contract, beyond predictability, just like a hailstorm ravaging the crops on a summer day. Roman legal solidarity could be brought to bear on anyone under the jurisdiction of Roman law, Roman citizen or stranger. French public law solidarity is emphatically a matter for and within the nation-state. Roman legal solidarity rested on a sophisticated set-up of two legal relations; one between the creditor, the debtor and sureties and another between the sureties and the debtor. At the level of principle, French legal solidarism was devoid of such precision, which one would need to hunt for in the capillaries of French social law. As we will see, these typological differences will be hugely important once we project them onto articulations of solidarity in EU law.

### Why Article 80 TFEU Is Deceptive

What difference does this conceptual history of solidarity make for our understanding of Article 80 TFEU? In the Roman law of obligation, solidarity presupposes a contract between a creditor and a debtor, backed up by one or more sureties. Under Article 80 TFEU, which actor would be analogous to a creditor, to a debtor and to a surety? And what would be the analogon to an agreement between creditor and debtor?

We have earlier concluded that the ‘principle of solidarity’ relates to the ordinary, daily operation of the CEAS, with the Dublin system at its core. Dublin creates imbalances in the distribution of asylum protection among the Member States. Could a stipulation on solidarity in primary law be sufficient as a matter of law to equalize the differences in protection burdens between Member States that the lawful operation of the Dublin system entails? On a benign reading, it could provide the analogon to the legal concept of obligation in solidum. However, nowhere is there a sign of the analogon of a legal debt needed to operate this obligation in solidum. We should recall that the differences between Dublin states’ protection obligations emerge as a matter of hard EU law, while the claim to offset them is political. The Roman law concept of solidarity is exactly not designed to handle moral–political debts but requires a legally valid debt to be applicable. This reading of the ‘principle of solidarity’ would quickly turn nonsensical. But let us verify: is there indeed no debt and no creditor in sight?

Actually, there could be. Human rights law and refugee law proscribe that Member States return persons in need of protection to states where they face risks or threats. These prohibitions of refoulement provide an asylum seeker with a right it may invoke against any of the Member States, once she is within the jurisdiction of a Member State. The European Convention on Human Rights and the 1951 Refugee Convention are the counterpart to the contract between creditor and debtor at the base of any relation of Roman law solidarity. And while the asylum seeker is a beneficiary of rather than a party to these treaties, the contracting parties have placed her in a position that gives her the power to insist on the performance of an agreed obligation on behalf of the creditor. The fact that Article 80 TFEU is silent on all this does not change the matter at hand. EU primary law has to be interpreted in conjunction with international legal obligations, and Articles 18 and 19 of the CFR protect rights that largely mirror non-refoulement obligations in international law. On this reading, we would have a debt (protection from refoulement) and a stand-in for the creditor (the asylum seeker). The states being bound by non-refoulement can be understood as being a group of debtors. What is lacking are the sureties characteristic for obligation in solidum. Refoulement provisions leave the distribution of burdens accruing from their application unregulated.^40^ A state confronted with an asylum claim could be seen to be in a position analogous to that of a debtor confronted with a claim. If that debtor denies the asylum seeker protection, there is no mechanism by which protection can be reliably claimed from a secondary state acting as the analogon to a surety.

There is one historical exception in the operation of EU asylum law where a system of sureties existed. We refer to the relocation mechanism that operated between 2015 and 2017 to alleviate the protective burden of certain frontline Member States by the legal obligation of other Member States to accept relocation of arrivals.^41^ While the fit of the analogy is not perfect, a frontline Member State as Malta could be seen as a debtor defaulting on the protection claims of arriving asylum seekers, with other Member States taking up the slack through their relocation obligation would act as sureties. The short-lived relocation scheme remains the only tangible approximation to Roman law solidarity. While the challenge to this scheme by Hungary before the CJEU remained unsuccessful, the system ultimately failed as there was no political will among Member States to continue its operation. If Roman law solidarity is but a brief exception in EU migration and asylum law, what is its rule?

Let us therefore inquire whether Article 80 TFEU shows traits of the French public law concept of solidarity. As we saw, the French concept does not presuppose an ex ante contract but is built on the tension between solidarity as a precondition for human survival and some factor threatening it—in the poignant words of the 1948 Constitution, a ‘calamity’. Article 80 TFEU seems to fit very well with this structure. After all, it is not only about asylum but about immigration and border control as well. The EU’s consistent position over the past decades has been that ‘illegal immigration’, an umbrella concept including most of the asylum seeking done in the Member States, is a threat that the Union and its Member States have to fight. Here is the analogon to a massive calamity that would call forth solidarity. And the very fact that solidarity is named over and above the ‘fair sharing of responsibility’ seems to suggest that solidarity is not fully exhausted in the fairness of sharing. The rationalism of sharing aside, there is something incomprehensible about solidarity just as there is something incomprehensible about *la Nation*. After all, neither Duguit nor any other French solidarity thinker of the nineteenth century can tell us why the social organism of solidarity is coextensive with the nation, and not with, say, a smaller or a larger social grouping.^42^

Here is our intermittent proposal for a reading of Article 80 TFEU. With the failure of the 2004 Treaty Establishing a Constitution for Europe in French and Dutch referenda, the EU was thrown back into being the piecemeal functional alliance on nation-statist grounds from which it had tried to emancipate itself for so long. If anything, the de-constitutionalization by referendum suggested that no social organism existed at a pan-European level. If you happened to be an EU policy maker and you were just denied a constitution by a couple of obstinate peoples, conjurations of solidarity would be the second best way forward. Hence, the EU gestured at the existence of a European social organism through a number of solidarity clauses in the Lisbon Treaty—among them, Article 80 TFEU. They suggest no less that a social organism does exist at Union level, over and above those implied in the nations of the Member States.

After reading Article 80 TFEU through the Roman legal concept and the French social concept of solidarity, are we compelled to conclude that it is devoid of meaning? Not quite. If we reinterpret it as a development of French public law solidarity into the form of an alliance, it makes sense. Out goes the search for an ex ante contract or an ex ante social organism. We just have to make good that a number of sovereign states decided to coordinate their efforts to defend themselves against an external calamity. It appears that we are experiencing just this right now. While there is wild disagreement on any solidaristic protection of refugees among Member States, they do agree on the necessity to protect external borders. If the threat that the thinkers of French positivist solidarity sought to avert were self-organizing French workers, the threat EU solidarity seeks to avert is the self-organization of migrants. Both forms of self-organization—that of nineteenth-century French workers and that of contemporary migrants to Europe—are read as systemic challenges to the political order of the day.

There remains a difference, though. Self-organizing workers in nineteenth-century France could be addressed in their capacity as *French* workers in the appeasement agenda that French solidarism formed part of. This appeasement went hand-in-hand with the repression of socialism in Europe at the time. Repression without appeasement was not an option, as the labour of French workers was needed for a rapidly industrializing economy. Self-organizing migrants, by contrast, need not be appeased. They are not part of a nation-statist project in Europe or the European project at large, and this is at least the folkloristic standard belief: there is no economic dependency on their labour. What remains is sheer repression. The object of that repression is the self-organization of a group of non-nationals, who organize their own travel and gain the status of an asylum seeker, however precarious. On the level of structure, this rhymes well with the Europeanization of solidarity thinking in the interwar period, which combined intra-European welfareism with a defensive stance against Bolshevism—a motive that would come into full bloom during the Cold War.

With those factors in mind, we will look into other solidarity provisions of the treaties in the next step and test whether they resonate with the Roman legal model, the French social model or,our third alternative by now, an alliance model. 

### Article 80 TFEU Compared to Other Solidarity Provisions

Among the ‘Common Provisions’ set out at the beginning of the TEU, Article 3.3 features solidarity language:

[The Union] shall promote economic, social and territorial cohesion, and solidarity among Member States.

The use of the verb ‘promote’ suggests that there is no hard obligation between creditor and debtor at work in this provision and that Roman legal solidarity is completely absent. Our reading in line with the logic of French social solidarity would be that there is no social organism at the EU level just yet, but to the extent the EU sees it emerging, it has to be promoted. Article 3.3 TEU would then only kick in if a ‘positivist’ analysis shows a European people to have emerged. That is quite a tall order indeed. In the alternative, solidarity in this provision is neither susceptible towards a Roman or a French interpretation and needs to be unlocked with a third interpretation.

Looking back at our analysis of Article 80 TFEU now, it appears that the same applies to that provision. It lacks the requisite interplay between obligation and entitlement under the Roman tradition. Neither can it be read in the light of French solidarism, because this would only entail a hard obligation on Member States and the Union if and only if European people have been shown to have emerged. It is unlikely, though, that this article has been drafted with such long foresight. To corroborate our point that the drafters simply were not on top of the concept they used, let us add an analysis of Article 42.7 TEU and of Article 222 TFEU in order to show that the use of solidarity language is equally misplaced there.

First is the mutual defence obligation of Article 42.7 TEU. There is no trace of the word ‘solidarity’ here, and we will soon explain why this is fully adequate. The provision reads as follows:

If a Member State is the victim of armed aggression on its territory, the other Member States shall have towards it an obligation of aid and assistance by all the means in their power, in accordance with Article 51 of the United Nations Charter. This shall not prejudice the specific character of the security and defence policy of certain Member States.

What strikes us first is that a calamity in the form of armed aggression is at work here. But if we were to look for a social organism that would act in solidarity when faced with such a calamity, the last sentence of Article 4.2 TEU would advise us that, ‘[i]n particular, national security remains the sole responsibility of each Member State’.

This suggests that Member States cannot be understood to express nineteenth-century French social solidarity at Union level in the area of national security. Therefore, we hold that Article 42.7 TEU is anything but a solidarity clause in the sense of the term reflected in Roman or French law. Reading it as an alliance clause or a clause of collective self-defence would seem to be appropriate. Among the OED entries on the term ‘alliance’, we find this one: ‘Combination for a common object, confederation, union offensive and defensive; especially between sovereign states’.^43^ While a combination of sovereign states for a common object might be very tight-knit in practice, it is still characterized by its focus on a single issue, as defence, or, indeed, migration and asylum. This is different from the idea of a people as a social organism, for whom anything calamitous could turn into a common object.

A comparison with the NATO provision on collective defence in Article 5.1 of the North Atlantic Treaty indicates that Article 42.7 TEU is a less articulate derivative of it:

The Parties agree that an armed attack against one or more of them in Europe or North America shall be considered an attack against them all and consequently they agree that, if such an armed attack occurs, each of them, in exercise of the right of individual or collective self-defence recognised by Article 51 of the Charter of the United Nations, will assist the Party or Parties so attacked by taking forthwith, individually and in concert with the other Parties, such action as it deems necessary, including the use of armed force, to restore and maintain the security of the North Atlantic area.

In a part of the Treaty addressing ‘The Union’s External Action’, the ‘Solidarity clause’ of Article 222 TFEU states the following in its first sentence:

The Union and its Member States shall act jointly in a spirit of solidarity if a Member State is the object of a terrorist attack or the victim of a natural or man-made disaster.

The remainder of the article offers what we might call procedural guidelines on how to orchestrate and coordinate prevention and assistance both by the EU and by its Member States. Under Article 222.4, the Council adopted a decision which offers even greater procedural granularity in this respect.

Is Article 222 really a solidarity clause? In the light of Article 42.7 TEU and Article 4.2 TEU, it cannot be, although the drafters confusingly named it so. National security is still a matter for the social organism of the Member States and not that of the Union. The external calamity of terrorism or natural disaster makes Member States enter a loose alliance with each other, which is something quite different from the concept of European solidarity.

There are two further examples that are neither about alliances nor about organic solidarity at the Union level: Article 122.1 TFEU on supply crises inter alia in the energy sector and Article 194 TFEU on energy policy provide for regulatory competency to support the functioning of the internal market. Union policy is to be articulated ‘in a spirit of solidarity between Member States’ in both provisions. We think that the reasons militating against the use of solidarity terminology are not as strong as in Article 222 TFEU, as energy market issues involve the regulatory competency of the Union. But as in Article 80 TFEU,^44^ the reference to solidarity is sheer decoration as long as we cannot make out a European social organism that would prop it up.

We have now reached a point where the conceptual history of solidarity and a lateral comparison of cooperation provisions in EU primary law point to the same conclusion: Article 80 should be understood as an alliance clause against the external threat that irregular migration, including that of asylum seekers, is perceived to be. The term ‘solidarity’ in it is a misnomer, unless we understand it as synonymous to defence against an external threat risking to turn into an internal one, just as Bolshevism must have been perceived by the Europeanist thinkers of the interwar period.

## Why Solidarity in the 2020 Pact Is Flawed

### The 2015 Emergency Relocation and Solidarity Disagreement

As it has been acknowledged in the literature, the 2015/2016 situation in Europe has been ‘first and foremost a policy crisis’ that highlights the ‘continuing failure of the CEAS’.^45^ EU policies responded mainly through deterrence (building fences, cooperation with Libyan authorities to block access to EU territories) and responsibility shifting to external partners (the EU-Turkey statement). In this context, European solidarity has been taking the form of financial assistance to third countries under a ‘protection closer to home’ mentality, much like the 1990s, yet without the burden-sharing component. This has rendered asylum in the EU and, by extension, solidarity a primarily exclusionary concept for the refugee. Although cooperation with third countries has been portrayed as a response to the ‘crisis’ and a way to ‘save lives’, it has been essentially a manifestation of the alliance constellation, defending the Union against an external calamity.

With regard to intra-EU solidarity, an emergency relocation mechanism based on Article 78 TFEU was designed to provide for provisional fair sharing of responsibility with countries located at the Union’s external borders. In particular, the Council of the EU adopted two relocation Decisions, on 14 and 22 September 2015,^46^ providing for the temporary and exceptional relocation, over 2 years, of 160,000 persons in clear need of protection from Italy, Greece and Hungary, to other Member States. Not all EU countries agreed to the decision. Two of them, Slovakia and Hungary, supported by Poland, challenged the legality of the second relocation decision before the CJEU for not conforming with the principles of necessity and proportionality.^47^

Compared to the ad hocism of the 1990s and the scheme’s poor implementation notwithstanding,^48^ the temporary institutionalization of relocation as a solidarity mechanism following the provisional suspension of the Dublin rules is a major development towards fairer responsibility sharing within the CEAS. As a measure of immediate relief to EU Member States under particular pressure, the Relocation Decisions have ensured access to protection to a significant number of applicants who would have otherwise been confined in countries of entry or might have engaged in irregular onward movement to other countries.^49^

However, due to the emergency nature of the decisions, they cannot be seen as fulfilling the rather strong demand of Article 80 TFEU for a solidarity that ‘governs’ EU asylum policies instead of serving as a short-term anti-crisis remedy. Furthermore, the asylum solidarity litigation shows precisely that there continues to exist a lack of a social organism, even when ‘a loss caused by the outside’, namely, increased refugee movements, is imminent. The conceptualization of solidarity as an undifferentiated set of obligations stemming from the emergency relocation mechanism, not attentive to the asymmetrical results of the 2015 refugee movements, was competing against the Visegrad group’s vision of flexible solidarity or effective solidarity.^50^ One cannot resist drawing a direct comparison here between the Roman law solidarity conception based on the ‘appearance’ of there existing a unit despite sovereign divisions and the disagreement on solidarity exposed by the 2015/2016 ‘refugee crisis’, during which concrete legal measures, enacted on the basis of solidarity, pushed the East–West divide in the EU to breaking point. Transposed to the Roman tradition, this is tantamount to the sureties turning recalcitrant once obligation in solidum has been contractually agreed.

The operationalization of solidarity through the emergency relocation mechanism also demonstrates that sharing schemes entail the honouring of particular obligations for countries at the receiving end; the ‘hotspot approach’ adopted to facilitate national authorities in Greece and Italy cope with operational tasks such as identification, registration, fingerprinting and return was precisely a way to ensure that solidarity goes hand in hand with (those countries’) responsibility. Representing more of an external border management measure which was hardly addressing reception or asylum demands, it ensured that responsibility does essentially lie with those Member States at the external borders.^51^

The disagreement on how solidarity should be given effect continued to haunt the EU states’ negotiations for the reform of the CEAS in the post-2015 era, leading to a deadlock concerning the proposal for a Regulation which would replace the Dublin III Regulation. In an effort to bring a new lease of life to what seemed to be an impossible task, the European Commission put forward the Pact, grounding it on ‘a new flexible and pragmatic approach’^52^ to solidarity in the CEAS as the basis for reforms which could eventually be agreed upon.

One might have expected that the COVID-19 pandemic would have urged radical reforms. For example, in view of the restrictions of movement at a global scale, some EU countries have made use of their sovereign right to grant protection on compassionate or humanitarian grounds to migrants who could not be safely removed (based on Article 6 para 4 of the Return Directive).^53^ Also the Commission could have drawn on instances where solidarity between the Member States did in fact work in cases of immediate demand (see, e.g. the relocation following the burning down of Moria). The reality has been that the Commission provided a patchwork of proposed measures on procedures, responsibility allocation, solidarity and partnerships. These build on pre-existing and flawed structures and do, essentially, institutionalize several controversial ad hoc deterrence measures suggested or implemented unilaterally or multilaterally by Member States as a response to 2015.^54^

### The Pact’s Flexible Approach and Its Extravagant Claims

The Pact as a legal construct is not new in the history of EU integration on migration and asylum. In 2008, the European Council adopted the European Pact on Immigration and Asylum^55^ which put a bold end to the possibility of the so-called mass regularizations of the status of migrants at the national level which had taken place in various Member States in the beginning of the 2000s. This has had a very tangible effect: asylum became the only possible means through which migrants could reside and work lawfully in a Member State.^56^ That made the harmonization of asylum rules and a common approach to borders and migration an imperative.

In particular, the 2008 Pact was seeking to reflect a celebration of the well-functioning and expansion of the Schengen agreement and free movement but also an appreciation of new challenges, stressing that the actions of one Member State will affect others in a more profound way than ever. The 2020 Pact has instead developed under the post-2015 climate of closed borders and a near obsession with management of the external borders as the precondition for going back to the regular application of Schengen. While the vocabulary on illegal migration control through return and border management is shared across the two Pacts, the 2008 Pact still employed the language of ‘a Europe of asylum’^57^ in which borders should not prevent access to protection. To the extent this expressed a modicum of solidarity between Europe and refugees, it no longer figured in the 2020 Pact. Another novelty is the 2020 Pact’s pre-screening and crisis mechanisms,^58^ based on an underlying binary logic of ‘international protection or removal’. In both cases though, there was no intention to reconsider the existing Dublin rules.

Maintaining the Dublin rationale, the 2020 Pact puts forward a proposal about an in-built flexible solidarity system, which expands the options for Member States to engage in sharing of responsibility, beyond the principle’s financial implications, as stated in Article 80 TFEU. Here, flexibility means engagement in relocation, return sponsorship and other—vaguely defined—capacity building measures. This constitutes an effort by the Commission to bring Member States’ divergent and even opposite visions of solidarity closer to each other. In fact, it entails a complex combination of voluntary pledges and mandatory contributions. The latter are to be determined through a distribution key calculated on the basis of Member States’ GDP and population, supplemented by ‘a mass correction mechanism’ to be activated when pledges under one specific form of solidarity (e.g. relocation or return sponsorship) fall short of the identified needs. This ‘pragmatic’ approach which, from a political standpoint may allow for ‘convergence despite disagreement’, leads to a complex arrangement at the expense of predictability and legal precision, which makes any comparison to the Roman law tradition of solidarity implausible.

Consider in particular the collectivization of responsibility for the downgrading of refugee rights in the proposed Regulation on crisis and force majeure in the field of asylum and migration. In accordance with its Article 3.1, a Member State turns to the Commission, claiming that a crisis situation is given. Say the Commission endorses its claim, leading to a curtailing of asylum seekers’ procedural rights under Article 4 of the proposed Regulation, dictated by the ‘exceptionality’ of the circumstances.^59^ In cases where the implementation of such crisis measures entail a violation of international human rights or refugee law, this begs the question of who bears responsibility for it: the Member State engaging in the actus reus of the violation, the Commission, all Member States involved or any combination of them.

As the Commission takes the decision under Article 4 of the proposed regulation, should we consider any ensuing violation to have taken place under its direction and control? If that can be argued, the EU could bear responsibility under Article 14 of the Articles on the Responsibility of International Organizations (ARIO). One precondition is that its decision left the Member State no other choice than to engage in the conduct amounting to a violation.^60^ Another precondition is that the act would have been internationally wrongful if committed by the EU, without the intermittent conduct of a Member State.^61^ These objections lead us back to Article 13 ARIO and Article 16 ARS, which addresses aid and assistance in the commission of an internationally wrongful act and an exploration of how the impugned conduct was shared between the EU and relevant Member States. Under the latter provisions, it will be rather difficult to delineate the exact responsibility of each of these actors, leading very likely to lacunae in the attribution of responsibility. The contrast to the clear-cut allocation of responsibilities under obligation in solidum could hardly be starker.

The Pact also fosters a concept of asymmetric solidarity, allowing Member States to pick and choose where and how to contribute in ways different from relocation. The proposed system is ‘responsive by design’, heavily reliant on the Commission deciding which contributions will be considered proportional and appropriate in case voluntary pledges are not sufficient. This raises a number of questions whose answer is postponed to a future point in time: what counts as a meaningful participation in sharing of responsibilities in financial and other terms? What kind of trade-offs will be legally acceptable in determining obligations? How is it guaranteed that no country is left alone in times of crisis if an agreement is not made as to what is fair and just? As mentioned in Sect. 2, implicating the European Commission or any other EU institution in defining deliverables is hardly different from the role assumed by the Council in the context of the Temporary Protection Directive. It could, thus, be argued that the EU is moving from a majoritarian interpretation of solidarity (unanimity via qualified majority) to an authoritarian one by means of Commission decision-taking.

Leon Duguit, one of the major exponents of French solidarist thought, suggested that the factual existence of an unwritten ‘social law’ was the base providing for solidarity. Without an everyday life supporting that social law, then, no solidarity. This is so even if the lawmaker provides for it in positive law. It is exactly here that the 2020 Pact is wanting: it lacks a self-evident community from which it emerges, and, by consequence, the concomitant bottom-up radicalism for solidaristic reforms. Unwritten solidarity evidently does not exist among Member States, as the political conflicts on emergency relocation corroborate, and the authors of the Pact are incapable of writing it into existence. Instead, solidarity in the Pact is informed by the pragmatism of the internal market and the Schengen area that requires a compromise. In particular, solidarity in the 2020 Pact is framed mainly as a matter where Member States, under the direction of the Commission, need to reach the best possible solution and less as a question of particular EU law obligation and of fundamental rights to be respected. Very much along the lines of a defence alliance, flexibility in the Pact comes at the expense of the asylum seeker who is commodified, traded and transferred between the EU Member States. In light of this, the rhetoric and function of solidarity (as relocation or return sponsorship) appears to be more of an *apology* for the Dublin rationale rather than the revision of an unfair system so direly needed. It is also doubtful if it could contribute to closing the existing implementation gap in so far as refugee preferences are not taken into account, border countries remain the gatekeepers, and the practicalities and politics of cooperation with third countries are taken for granted.

This ‘pragmatic take’ on solidarity obscures any discussions on solidarity at a meta-level.^62^ How is the future of the CEAS envisioned and what is it that solidarity should achieve? As argued earlier, the Commission builds its proposals on a non-existing spirit of Union solidarity and on a non-existing peoples. As a matter of fact, the Pact is based on EU Member States’ disagreement on solidarity which is expected to not go away for as long as the discussion of these meta-questions and competing visions are postponed to a future time. In this respect alone, the Pact honours the tradition of French nineteenth-century solidarism, which, with a fair dose of dirigisme, steered clear from the overarching conflict besetting French society during the Third Republic.

## Conclusions

The debate on the 2020 Pact provided us with a springboard, and we brought the question of solidarity in EU migration and asylum law into conversation with two dominant historical conceptualizations of solidarity so as to explore the extent to which they resonate with each other. The question driving the analysis was why solidarity has not worked according to expectation. From the 1990s onwards, a normative heritage built up around the Dublin system, with the guarding of external borders allotted primacy over the sharing of protection obligations. The ensuing path dependency on Dublin unmitigated by burden-sharing severely constrained the significance of the primary law concept of solidarity introduced in 2009. We have shown that there is a structurally conditioned mismatch between the expectations created by the use of solidarity terminology and the reality of EU law. The conceptual heritage of solidarity as it plays out in Roman private law creates the expectation of a predictable and legally enforceable scheme of responsibility allocation, while French public law solidarism creates expectations of a social fact of mutuality manifesting ex nihilo at the foundation of EU law. The reality these expectations meet is not only the vagueness of article 80 TFEU, which is set in sharp relief by the precision of Roman obligation in solidum. It is also the absence of the social fact of solidarity within a relevant population, elementary for the French tradition of solidarity thinking. Article 80 TFEU can be understood if we combine two observations. First, Article 80 TFEU makes sense once we see the rationale of French solidarism in it; both French solidarism and its EU law inheritor being part of politics that counters the self-organization of a population perceived as a threat to the established order. Second, that reading is reinforced once we learn that French solidarism gave way to interwar Europeanist tradition inspired by it and emphasizing that Europe must be defended against an infiltration by an external threat (Bolshevism, as it were).

Drawing on the history of EU asylum and migration policy since its intergovernmental beginnings, we respond in particular to the questioning of the 2020 EU Pact, pointing out what these questions miss or take for granted. We suggest that the EU is de facto going back to old practices of disintegration through’common but differentiated responsibility’ without sufficient predictability of contributions materializing in situations of need. Compared to the failure of agreeing on an equitable sharing of Bosnian refugees in the 1990s, no progress has been made. In particular, the history of solidarity in the CEAS demonstrates that solidarity is invoked when the law, with the Dublin system as its central tenet, has failed. Article 80 TFEU juridifies a communitarian–instrumentalist concept of alliance devised to defend borders and manage migration. In an effort to ‘give effect to’ Article 80 TFEU, the Pact focuses on obligatoriness and flexibility, with the new solidarity mechanism prescribing obligations with a quality of abstraction, as the promise of solidarity is forever postponed to the future. Just as nineteenth-century solidarism once did with regard to organizing French workers, EU asylum solidarity is seeking to immobilize migrants.

## Footnotes

1 European Commission, Communication from the Commission to the European Parliament, the Council, the European Economic and Social Committee and the Committee of the Regions on a New Pact on Migration and Asylum, COM/2020/609 final.

2 European Commission, Communication from the Commission to the European Parliament, the Council, the European Economic and Social Committee and the Committee of the Regions on a New Pact on Migration and Asylum, COM/2020/609 final, 2.

3 See, among others, Carrera and Geddes (2021).

4 See European Commission, Proposal for a Regulation of the European Parliament and of the Council on asylum and migration management and amending Council Directive (EC) 2003/109 and the proposed Regulation (EU) [Asylum and Migration Fund] COM/2020/610 final. The proposal for an Asylum and Migration Management Regulation (RAMM) offers various alternatives for states to fulfil their solidarity obligations (Part IV Solidarity, Articles 45–60), which has revived discussions about two-speed Europe, deflectors and asymmetry.

5 Council Decision (EU) 2015/1523 of 14 September 2015 establishing provisional measures in the area of international protection for the benefit of Italy and of Greece 2015 OJ L239/146 and Council Decision (EU) 2015/1601 of 22 September 2015 establishing provisional measures in the area of international protection for the benefit of Italy and Greece 2015 OJ L248/80.

6 CJEU, C-643/15 and C-647/15, Slovakia and Hungary v Council, 6 September 2017, paras 291-293.

7 On this, see Brouwer et al. (2021).

8 For an extensive discussion on burden-sharing practices during the Bosnian and Kosovo crisis and their distributive effects, see Noll (2000) and Noll (2002).

9 For a detailed analysis, see Betts et al. (2017), 74. See also Koser et al. (1998).

10 Resolution on the Harmonization within the European Community of Asylum Law and Policies (adopted 15 November 1992) Doc. No. A3-0337/92. OJ 1992, C 044/106; Resolution on the General Principles of a European Refugee Policy (adopted 19 January 1994) Doc. No. A3-0402/93. OJ 1994, C 044/106; Council Resolution of 25 September 1995 on Burden Sharing with Regard to the Admission and Residence of Displaced Person on a Temporary Basis, (95/C 262/01).

11 See Council Resolution of 30 November 1992 on a Harmonized Approach to Questions Concerning Host Third Countries, Council Resolution of 30 November 1992 on Manifestly Unfounded Applications for Asylum (London Resolutions). For an analysis of safe third country rules and their implications for refugee protection, see among others, Costello (2016); Moreno-Lax 2015; Freier, Karageorgiou and Ogg (2021).

12 See, among others, Guild (2006), 635–6; Lavenex (2001), 98–9.

13 Treaty of Amsterdam amending the Treaty on European Union, the Treaties establishing the European Communities and certain related acts [1997], art 73 k, 2(b).

14 European Council, 15 and 16 October 1999 Tampere, Presidency Conclusions, 16/10/1999 – Nr: 200/1/99, 16.

15 Commission of the European Communities, Commission Staff, Revisiting the Dublin Convention: developing community legislation for determining which Member State is responsible for considering an application for asylum submitted in one of the Member States, SEC (2000) 522, para 20.

16 Ibid, para 19. As an example of a ‘clear and workable’ alternative, the Commission did refer to the approach of allocating responsibility according to where the first asylum claim is lodged yet, it underscored that such an alternative ‘implies though a choice between objectives which can be recognised as equally important’. See para 59.

17 Ibid, para 30.

18 European Commission, proposal for a joint action concerning solidarity in the admission and residence of beneficiaries of the temporary protection of displaced persons COM/98/0372 final/2, OJ C 268, 27 August 1998, arts 3 and 4.

19 Council of the European Union, Council Decision No 2000/596/EC of 28 September 2000 establishing a European Refugee Fund, OJ L252/12.

20 The Directive was criticized for the absence of predefined commitments for solidarity. Reliance on the Council to agree on their existing a situation that qualifies as ‘mass influx’ was feared as a prolonged process to be avoided.

21 See European Council, The Hague Programme: Strengthening Freedom, Security and Justice in the European Union (2005/C 53/01) I. Introduction where it is stated that ‘The security of the European Union and its Member States has acquired a new urgency, especially in the light of the terrorist attacks in the United States on 11 September 2001 and in Madrid on 11 March 2004. The citizens of Europe rightly expect the European Union, while guaranteeing respect for fundamental freedoms and rights, to take a more effective, joint approach to cross-border problems such as illegal migration, trafficking in and smuggling of human beings, terrorism and organised crime, as well as the prevention thereof. Notably in the field of security, the coordination and coherence between the internal and the external dimension has been growing in importance and needs to continue to be vigorously pursued’.

22 Council Regulation (EC) No 2007/2004 of 26 October 2004 establishing a European Agency for the Management of Operational Cooperation at the External Borders of the Member States of the European Union OJ L 349.

23 Joly (1996), 75.

24 Meeting of Ministers Responsible for Immigration, Summaries and Communiques, October 1986-June 1993, Council of the European Communities General Secretariat Press Release, London 30 November 1992 10518/92 (Presse 230- G).

25 Charter of Fundamental Rights of the EU, 2000/C 364/01 ch IV. ‘Conscious of its spiritual and moral heritage, the Union is founded on the indivisible, universal values of human dignity, freedom, equality and solidarity’.

26 European Council, The Stockholm Programme. An Open and Secure Europe Serving and Protecting Citizens, (2010/C 115/01), 5.

27 Noteworthy samples from the past decade include Sangiovanni 2013 (asking what principles of social solidarity ought apply between states and citizens of the emerging Euro[ean polity), Sangiovanni 2016 (asking in what sense, if any, social justice is best understood as a demand of solidarity), Kolers 2016 (developing a deontological theory grounded in equity), Eleftheriadis 2020 (arguing that the substance of EU law is international, not constitutional, discussing solidarity in terms of fairness) and Dziedzic 2022 (exploring solidarity and the CEAS from the perspective of collective action).

28 Zimmermann (1996), 2.

29 Zimmermann (1996), 10.

30 By way of illustration, the response to this secondary question might be *cessio legis*, a transfer of obligations from the creditor to that surety making the payment. That surety can then turn to the remaining creditors to seek payment.

31 Zoll (2000), 18–19.

32 ‘Solidarism could not escape the accusation that it was fraternity dressed up in scientific clothes. However, it was popular because many aspirations—socialist, aesthetic and Christian—found some echo in its teachings. … It had more than a suggestion of being designed to steal the thunder of the socialists. It was more or less contemporaneous with William II’s new course in German politics, in which Christian socialism was aimed at winning the workers away from revolution: it could be called a lay version of it’. Zeldin (1993), 659.

33 In the 1891 encyclical Rerum Novarum, Pope Leo XIII aptly stated that ‘the more that is done for the benefit of the working classes by the general laws of the country, the less need will there be to seek for special means to relieve them’. This resonates with the contemporaneous conception of la dette social, the social debt, and it is fair to assume that the ‘special mean’ alluded to would otherwise be sought through socialism.

34 Grimm (1973), 38, crediting Fouillée for being the founder of solidarism.

35 Duguit, as quoted in Grimm (1973), 42, at note 59.

36 Duguit, as quoted in Grimm (1973), 42, at note 59.

37 Duguit, Scelle.

38 White (2016), 29.

39 Aristide Briand’s project for the creation of a European Union was announced in 1929 at the Assembly of the League of Nations. Until the start of World War II, this federative idea was discussed mainly in connection to a system of security. On this, see Papadaki (2012), 221–231.

40 The Conference of Plenipotentiaries would contend itself with recommending ‘that Governments continue to receive refugees in their territories and that they act in concert in a true spirit of international cooperation in order that these refugees may find asylum and the possibility of resettlement’ in the Final Act to the 1951 Convention.

41 See Sect. 4.1.

42 In an amazing review of the literature as it presented itself at the outset of the twentieth century, Célestin Bougle identified original sin as the common base of conceptualizations of solidarity (Bouglé (1906)). That would throw a different light on the personal referent of the solidarity question. Within Christianity, the fall is an experience common to all mankind as the progeny of Adam and Eve. It would run counter to French ideas of the nation as the ultimate referent of solidarism. The argument on original sin is intricate, giving solidarity its conceptual contours through a negative referent: the absence of community with god enacted in the eviction from paradise.

43 ‘alliance, n.’. OED Online. September 2021. Oxford University Press. www.oed.com/view/Entry/5290 (accessed February 4, 2021).

44 On the link between the principle of solidarity in asylum policy and that in energy policy see CJEU, C-848/19 P Federal Republic of Germany v Republic of Poland, European Commission 15 July 2021, paras 42-44 and Advocate General Opinion 18 March 2021, paras 68-73.

45 See, e.g. Chetail (2016) and den Heijer et al. (2016).

46 Supra note 5.

47 Supra note 6.

48 As of September 2017, 27,695 people had been relocated from Greece and 8,451 from Italy based on the European Commission, ‘Thirteenth report on relocating and resettlement’ COM (2017) 330 final.

49 ECRE, ‘Relocation of asylum seekers in Europe: a view from receiving countries’ May 2018.

50 See Joint Statement of the Heads of Governments of the V4 Countries available at https://www.visegradgroup.eu/calendar/2016/joint-statement-of-the-160919 (accessed October 29, 2021); EU Council, Slovak Presidency non-paper ‘Effective Solidarity A Way Forward on Dublin Revision available at https://www.statewatch.org/media/documents/news/2016/nov/eu-council-slovak-pres-non-paper-dublin-effective-solidarity-11-16.pdf (accessed October 29, 2021).

51 Further on this, see Karageorgiou (2019).

52 Press statement by President von der Leyen on the New Pact on Migration and Asylum, 23 September 2020.

53 See the example of Portugal https://ec.europa.eu/migrant-intergration/news/portugal-more-than-356-000-immigrants-provisionally-legalised-during-covid-19-pandemic (accessed October 29, 2021).

54 For a detailed analysis, see Carrera and Geddes (2021) and Brouwer et al. (2021).

55 European Union: Council of the European Union, European Pact on Immigration and Asylum, 24 September 2008, 13,440/08.

56 By way of example, in the mid-2000s, a significant number of undocumented migrants in Greece including Albanians who did not qualify as refugees, mindful of the fact that the Greek asylum system suffered from long delays in delivering decisions, filed asylum applications merely to access the rights that being an asylum seeker implied. When their applications were examined in substance some 10 years after the original application, authorities regularly rejected international protection claims. However, many of these applicants were granted permission to stay on humanitarian grounds.

57 Ibid European Pact on Immigration and Asylum 2008, 4.

58 European Commission, Proposal for a Regulation of the European Parliament and of the Council introducing a screening of third-country nationals at the external borders and amending Regulations (EC) No. 767/2008, (EU) 2017/2226, (EU) 2018/1240 and (EU) 2019/817 COM/2020/612 final and European Commission, Proposal for a Regulation of the European Parliament and of the Council addressing situations of crisis and force majeure in the field of migration and asylum, COM(2020) 613 final.

59 European Commission, Proposal for a Regulation of the European Parliament and of the Council introducing a screening of third-country nationals at the external borders and amending Regulations (EC) No. 767/2008, (EU) 2017/2226, (EU) 2018/1240 and (EU) 2019/817 COM/2020/612 final and European Commission, Proposal for a Regulation of the European Parliament and of the Council addressing situations of crisis and force majeure in the field of migration and asylum, COM(2020) 613 final.

60 As the ILC commentary to Article 14 suggests, ‘The assumption is that the State or international organization which is the addressee of the decision is not given discretion to carry out conduct that, while complying with the decision, would not constitute an internationally wrongful act’. United Nations, Report of the International Law Commission, GAOR 61st sess., suppl. no. 10, New York 2009, 88.

61 Article 14.2 ARIO.

62 Further on pragmatism and solidarity, see Karageorgiou (2021).
